# Cases of the Angina Trachealis, with the Mode of Cure, in a Letter to William Hunter, M. D. &c. &c.

**Published:** 1781-04

**Authors:** 


### LONDON MEDICAL JOURNAL,

For APRIL 1781.

### SECTION I.

### BOOKS.

I. *Cases of the Angina Trachealis, with the Mode of Cure, in a Letter to* William Hunter, M. D. *&c. &c. By* Richard Bayley, *Surgeon. To which is added, a Letter from* Peter Middleton, M. D. *to the Author.* 8vo. New York, 1781, 23 pages.

THE disease here treated of has been distinguished by various appellations. By Dr. Home, it is called the *croup*; by Dr. Cullen, *cynanche trachealis*; by Dr. Michaelis, *angina polyposa membranacea*; by Dr. Bard, *angina suffocativa*; and by Dr. Johnston, *angina trachealis*, which is the name adopted by the author of the work before us, who informs us, that in New England it is vulgarly

 called called *the bladder in the throat*, and in Jerſey and Penſylvania, the *hives*.

Mr. Bayley conſiders it as an inflammatory diſeaſe, and is of opinion that the moſt deſperate caſes may yield to repeated bleedings *ad deliquium* from the jugulars, and the free uſe of tartar emetic and other evacuants, provided a large bliſter is applied at the ſame time to the larynx and aſpera arteria, and the mucus filling up the ramifications of the bronchia is thrown up by the action of vomiting.

The firſt caſe that came under our author's inſpection was in 1774. The patient, a fine boy four years old, well made, but ſubject to aſthma, was obſerved to droop ſeveral days previous to the application of his friends for medical aſſiſtance, and in the night his indiſpoſition and cough affected him much more ſeverely. He was bled, a large bliſter was applied to the throat, and calomel and antiſeptics were adminiſtered internally; the patient died, however, within thirty-ſix hours from the firſt fit of ſtrangulation. Soon after a ſimilar caſe occurred, and proved equally fatal. On diſſection, the fauces were found covered with an aſh-coloured mucus of very little conſiſtence, which, by applying the ſmalleſt force, could eaſily easily be removed: the pendulous palate was enlarged and livid, and the whole of the trachea lined with a membrane of a whitish colour, and of such tenacity as to require a considerable force to pull it asunder; after entering the bronchia it changed its consistence, and became a glary mucus.

Shortly after this dissection, our author was called in consultation with Dr. Van Vleck, to visit a child which had been attacked three days before with a putrid sore throat. The patient died on the seventh day. During the last stages of this disease, the child's breathing was much interrupted, and in expiration the voice was very hoarse and loud, though the face was not swoln, nor the jugulars distended. On dissection, the fauces exhibited the appearance of one continued ulcer; the tonsils were totally destroyed; the velum pendulum palati was changed to a mere suspended slough; but the larynx and trachea were free from every appearance of disease.

After reflecting on the manner of this child's death, and comparing its circumstances with the preceding cases, Mr. Bayley thought it probable that the angina trachealis had not been sufficiently investigated by practitioners, who may have miſtaken the like inſtances of *hoarſe noiſe* in the *putrid ſore throat*, for the louder hoarſeneſs and *ſhrill voice*, which in a great meaſure are characteriſtic of the *angina trachealis*. The truth of this remark he thinks confirmed, from his having obſerved that thoſe writers who have made moſt mention of this diſeaſe (Drs. Bard and Home excepted) have favoured us only with an opinion of its nature, without offering any corroborating proofs from diſſection. The ſudden death of many who have been affected with this complaint, added to the caſes related by the writers juſt now mentioned, convinced our author that a more determined mode of treatment was requiſite in a diſeaſe which appeared to be altogether inflammatory. He obſerves, that the ſloughs in theſe caſes are not in conſequence of diſeaſed parts, being merely a concreted mucus which may be eaſily removed, and when that is done the membrane beneath it is found entire. He remarks, likewiſe, that the hoarſe ſound (with ill-ſcented breath) which in ſome inſtances attends the ulcerous ſore throat, will be found for the moſt part in thoſe caſes which attack with ſome degree of inflammation, where the tonſils are in conſequence enlarged, and the pendulous palate much much tumefied. In ſuch caſes there will be a conſiderable collection of mucus in the pharynx, preſſing upon the epiglottis and rimula of the larynx, whence will ariſe a partial difficulty in the air's paſſing to and from the lungs, with a quickened reſpiration and red cheeks, accompanied with more or leſs noiſe.

Having acquired theſe ideas of the nature of the diſeaſe, our author reſolved to treat it accordingly; and having been called to a child who was remarkably plethoric, ſhort necked, and of a dark complexion, and whoſe difficulty of breathing, hoarſe ſound, and ſhrill voice did not admit the leaſt doubt of the nature of the complaint, he recommended bleeding *ad deliquium*. On recovering from this ſtate, the patient puked a great quantity of phlegm, ſome of it viſcid, and ſome without conſiſtence, but of a very offenſive nature. The vomiting was encouraged with warm water, after which his breathing became leſs laborious; the noiſe accompanying it abated conſiderably, and his countenance changed remarkably for the better. A bliſter was immediately applied, large enough to cover the larynx, aſpera arteria, and part of the cheſt; emetic tartar in doſes ſufficient to keep up a continued nauſea was adminiſtered, and now and then increaſed, ſo as to excite vomiting. Calomel was given as an evacuant, and clyſters to promote its operation. Within a few hours after this mode of treatment was adopted, the child began to draw his breath with apparent freedom, and by a perſeverance in the antiphlogiſtic plan, and promoting a diſcharge by means of the bliſter, the patient recovered.

Our author ſucceeded in another caſe nearly ſimilar to the above, and by the ſame methods.

From 1774 to 1779, no opportunities occurred to our author of treating this diſeaſe; but in the mean time he had profited in his obſervations by diſſecting ſeveral who died of the complaint, all of which confirmed his opinion of the inflammatory nature of the diſeaſe, and juſtified the mode of treatment he had adopted. Among others, he opened a boy of about fourteen years of age, who had lived eight days in extreme miſery. In this ſubject, the root of the tongue, the pendulous palate and fauces, were covered with a much thicker and browner coat than uſual; but upon removing this new-formed membrane, the parts beneath it were found found entirely free from every appearance of difeafe.

In Auguft 1779, the author was called to a child who had paffed a reftlefs night, and for a few minutes was threatened with fuffocation; the next morning it was fo much better as to run out into the ftreet, though its voice was hoarfe. About noon it was attacked with additional violence. It was bled *ad deliquium* from the arm; emetic tartar, a blifter, and calomel were had recourfe to as in the former cafes, and the patient recovered.

A fourth cafe is related of a child two years old, who after being apparently recovered from the meafles, was attacked with cough, exceffive difficulty of breathing and hoarfenefs, accompanied with a fhrill found very diftreffing to every ear. When Mr. Bayley faw the patient his face was enlarged, and the jugulars greatly diftended. An attempt was made to bleed the child in the arm; but as only four ounces of blood could be procured in this way, one of the jugulars was opened, and the patient bled *ad deliquium*. Emetic tartar was given in naufeating dofes, and a large blifter applied to the throat. The next day the child was fomewhat relieved, having paffed a tolerable night, but the hoarſeneſs and dyſpnœa were ſtill conſider-able. The patient was again bled *ad deliquium* from the jugular, a purgative was given to procure ſtools, and the uſe of the emetic tartar continued. On the ſucceeding day the child puked twice a thin glary matter, with ſome mucus, the hoarſe ſound was ſcarcely perceptible, and the cough not troubleſome. From this date the ſymptoms grew more and more favourable, and by a continuance of the ſame treatment the child perfectly recovered.

Dr. Middleton, in a letter to Mr. Bayley inſerted at the end of this little work, obſerves, that when he firſt came to New York in 1752, he found complaints of the throat not unfrequent; but as they were generally conſidered as having a putrid tendency, antiſeptics were the remedies commonly employed in preference to all evacuants, except perhaps emetics. Being a ſtranger to the climate and its diſeaſes, he for ſome time acquieſced in theſe notions; but it was not long before he had an opportunity of being convinced that the diſeaſes of the throat had not been properly diſtinguiſhed, and that the croup was truly of an inflammatory nature. He mentions ſeveral caſes in which copious bleeding in the jugular, with the uſe of bliſters, bliſters, and evacuants ſucceeded, and ſpeaks of others that proved fatal where thoſe remedies, eſpecially venæſection, were neglected or objected to. In two diſſections of children who died of this diſeaſe, he met with nearly the ſame appearances as thoſe deſcribed by Mr. Bayley. In one, no ulcers were to be ſeen, or any remarkable lividity; but the glands were ſo much tumefied, as to have occaſioned the child's death by ſuffocation. In the other he obſerved no remarkable tumefaction of the glands of the throat, no lividity, nor any appearance of an ulcer. On opening the trachea, he found it lined with a whitiſh circular membrane of about the thickneſs of very fine white paper, which adhered to the trachea by a viſcid mucus underneath, by means of which there was ſome ſpace between it and the trachea. The grand bronchial diviſions were filled with this mucus.

Dr. Middleton concludes with remarking, that this diſeaſe is totally diſtinct from the malignant ſore throat, and that it is not contagious as ſome have ſuppoſed it to be. He does not preſume to ſay that it never is combined with the malignant ſore throat; but he aſſerts, that of the many caſes he has attended, he has never met with a ſingle inſtance where they were united.

